# Non-monotonic changes in clonogenic cell survival induced by disulphonated aluminum phthalocyanine photodynamic treatment in a human glioma cell line

**DOI:** 10.1186/1479-5876-8-43

**Published:** 2010-04-30

**Authors:** Seema Gupta, Bilikere S Dwarakanath, K Muralidhar, Tulay Koru-Sengul, Viney Jain

**Affiliations:** 1 Institute of Nuclear Medicine and Allied Sciences, Brig. S. K. Mazumdar Road, Delhi-110054, India; 2 Department of Radiation Oncology, University of Miami, Miami, FL 33136, USA; 3 Sylvester Comprehensive Cancer Center, University of Miami, Miami, FL33136, USA; 4 Department of Zoology, University of Delhi, Delhi-110007, India; 5 Department of Epidemiology and Public Health, University of Miami, Miami, FL33136, USA; 6 Eco-Development Foundation, New Delhi 110001, India

## Abstract

**Background:**

Photodynamic therapy (PDT) involves excitation of sensitizer molecules by visible light in the presence of molecular oxygen, thereby generating reactive oxygen species (ROS) through electron/energy transfer processes. The ROS, thus produced can cause damage to both the structure and the function of the cellular constituents resulting in cell death. Our preliminary investigations of dose-response relationships in a human glioma cell line (BMG-1) showed that disulphonated aluminum phthalocyanine (AlPcS_2_) photodynamically induced loss of cell survival in a concentration dependent manner up to 1 μM, further increases in AlPcS_2_concentration (>1 μM) were, however, observed to decrease the photodynamic toxicity. Considering the fact that for most photosensitizers only monotonic dose-response (survival) relationships have been reported, this result was unexpected. The present studies were, therefore, undertaken to further investigate the concentration dependent photodynamic effects of AlPcS_2_.

**Methods:**

Concentration-dependent cellular uptake, sub-cellular localization, proliferation and photodynamic effects of AlPcS_2 _were investigated in BMG-1 cells by absorbance and fluorescence measurements, image analysis, cell counting and colony forming assays, flow cytometry and micronuclei formation respectively.

**Results:**

The cellular uptake as a function of extra-cellular AlPcS_2 _concentrations was observed to be biphasic. AlPcS_2 _was distributed throughout the cytoplasm with intense fluorescence in the perinuclear regions at a concentration of 1 μM, while a weak diffuse fluorescence was observed at higher concentrations. A concentration-dependent decrease in cell proliferation with accumulation of cells in G_2_+M phase was observed after PDT. The response of clonogenic survival after AlPcS_2_-PDT was non-monotonic with respect to AlPcS_2 _concentration.

**Conclusions:**

Based on the results we conclude that concentration-dependent changes in physico-chemical properties of sensitizer such as aggregation may influence intracellular transport and localization of photosensitizer. Consequent modifications in the photodynamic induction of lesions and their repair leading to different modes of cell death may contribute to the observed non-linear effects.

## 1. Background

Photodynamic therapy (PDT) involves excitation of sensitizer molecules by visible light in the presence of molecular oxygen, thereby generating reactive oxygen species (ROS) through electron/energy transfer processes. The reactive oxygen species, such as singlet oxygen and hydroxyl radicals thus produced can cause damage to both the structure and the function of the cellular constituents resulting in cell death. Photodynamic effects resulting either in apoptotic, mitotic and/or necrotic cell death depend on the nature of the photosensitizer, cell type and the cellular targets for photosensitization, concentration and intracellular localization of the sensitizer [1,2], the incubation conditions and the light dose [2-4]. Clinical formulation of hematoporphyrin derivative (HpD), commercially known as photofrin II (PF-II) is being used presently for the treatment of esophagus, bladder and lung cancers in several countries [5]. However, a complex chemical composition, lower molar absorption coefficient in the red region, unfavorable intracellular localization and skin photo-toxicity limit the therapeutic applications of HpD [6]. Therefore, attempts have been made to overcome the limitations by the use of a) better sensitizers and b) strategies that target the sensitizer preferentially to the tumor and also to the more sensitive intracellular sites. Towards this end, second generation water soluble sensitizers such as phthalocyanine (Pc) derivatives are being widely investigated for their photodynamic effects [7,8]since these sensitizers are characterized by a more efficient absorption of therapeutically useful light wavelengths, especially in the 650-800 nm spectral range [9], permitting light penetration into tissues to almost twice the depth of that achieved using porphyrin PDT enabling photodynamic treatment of remote tissues [8,10,11]. Also, Pcs have low absorption of light at other wavelengths, thus lowering the risk of skin photosensitivity. The sulphonated derivatives of phthalocyanine have undergone extensive investigations *in vitro *and *in vivo *showing significant phototoxicity [7,9,10,12]. Results from the present investigations of dose -response relationships in a human glioma cell line (BMG-1) show that disulphonated aluminum phthalocyanine (AlPcS_2_) photodynamically induces loss of cell survival (assayed by clonogenicity) in a concentration dependent manner up to 1 μM, while further increases in AlPcS_2 _concentration (>1 μM), decreases the photodynamic efficiency. Considering the fact that for most photosensitizers only monotonic dose-response (survival) relationships have been reported [13], this result was unexpected. The non-monotonic dose-response characteristics of a photosensitizer could have interesting implications for PDT. The present studies were, therefore, undertaken to further investigate the concentration dependent photodynamic effects of AlPcS_2 _and to gain insight into the mechanisms underlying these effects.

## 2. Materials and methods

### 2.1 Tumor cell lines

Human cerebral glioma cell line (BMG-1; DNA index = 0.95; wild-type p53), established from a mixed glioma [14] was used in the present studies.

Monolayer BMG-1 cells were grown in DMEM with 5% fetal calf serum (FCS), penicillin (100 units/mL), streptomycin (50 μg/mL) and nystatin (2 μg/mL). Stock cultures were passaged every third day after harvesting the cells with 0.05% trypsin and seeding 8 × 10^3 ^cells/cm^2 ^in tissue culture flasks to maintain the cells in the exponential phase. All experiments were carried out with exponentially growing cells.

### 2.2 Chemicals

Disulphonated aluminum phthalocyanine (AlPcS_2_) was prepared and characterized in INMAS, Delhi and consisted of a mixture of isomers with sulphonic groups in both adjacent and opposite positions [15]. Hank's Balanced Salt Solution (HBSS), Dulbecco's modified Phosphate Buffered Saline (PBS), Dulbecco's Modified Eagle's Medium (DMEM), fetal calf serum (FCS), (N-[2-Hydroxyethyl] piperazine-N'-(2-ethanesulfonic acid]) (HEPES) buffer, propidium iodide (PI), 4,6 diamidino 2-phenyl indole (DAPI), Ribonuclease-A (RNase-A) and trypsin were obtained from Sigma Chemical Co., USA.

All other chemicals used in the present study were of analytical grade from BDH, Glaxo laboratories (Qualigens), SRL, and E-Merck, India.

### 2.3 Absorption and emission (fluorescence) spectroscopic measurements in cell suspension

Cells were trypsinized, counted and incubated in dark for various time intervals (1, 2, 4 h) with various concentrations of AlPcS_2 _(1-10 μM) in HBSS at 37°C. After incubation of cells in HBSS, both the absorption and fluorescence spectra (exc. 610 nm; em. 625-800 nm) of cells and supernatant before and after washing were obtained independently (Model JY3C, Jobin Yvon, France). Cellular uptake was calculated using standard calibration curves of photosensitizer in HBSS.

### 2.4 Subcellular localization using fluorescence image analysis system

Intracellular localization of AlPcS_2 _was studied by fluorescence microscopy using image analysis system (Olympus, BX60, Japan) equipped with a monochrome CCD camera (Gründig, FA87, Germany).

Cells were grown on cover-slips for these studies. After incubation with AlPcS_2_, cover-slips were washed in PBS, mounted on slides and examined under the fluorescence microscope using UV excitation filter (300-400 nm) and emission recorded in 400-800 nm region of the spectrum. Images were acquired and stored in digital computer (166 MHz) and analyzed using the software provided by Optimas Corporation, USA.

Cytoplasmic and nuclear localization of the sensitizer was estimated by analyzing the images using area morphometry by marking the appropriate regions of interest (ROI). For uptake measurements also, area morphometry that provides the average amount of the photosensitizer in the whole selected area, was used [16].

### 2.5 Photodynamic treatment

Cells growing as adherent monolayer cultures were incubated in HBSS at 37°C for 2 h with varying concentrations of AlPcS_2 _(0.25-10 μM). Post-incubation, cells were washed with HBSS and exposed to red light (Power = 3 W/cm^2^) from a high power (1000 W) Xenon arc lamp (Oriel, USA), using an optical filter (cut off at 610 nm) with the petridishes placed on ice. Optical power at the cell surface was measured using radiometer (Model 1400 A, International Radiometer, USA) having a detector head (SL021/FQ) with a flat response between spectral range 400-1000 nm. The cells were euoxic with oxygen levels provided by dissolved oxygen in the media. Cells were incubated for further 2 h at 37°C in HBSS before assay of cell response to treatment.

### 2.6 Cellular response to photodynamic treatment

#### 2.6.1 Clonogenic survival assay

Nearly 150 cells were plated in growth medium (DMEM + 10% FCS) after the treatment (as described above) and incubated in dark under humidified CO_2 _(5%) atmosphere at 37°C for 8-10 days to allow colony formation. Colonies were fixed with methanol and stained with 1% crystal violet. Colonies having more than 50 cells were counted and plating efficiency (PE) and surviving fraction (S.F.) were calculated.

#### 2.6.2 Cell proliferation kinetics

After photodynamic treatment, attached monolayer cells were incubated in growth medium, harvested and counted (using hemocytometer) after varying intervals of time. Floating cells were collected separately before harvesting attached cells by trypsinization. Flow-cytometric measurements of cellular DNA contents were performed with the ethanol (70%) fixed cells using the intercalating DNA fluorochrome, propidium iodide (PI) as described earlier [17]. Measurements were made with a laser based (488 nm) flow-cytometer (Facs Calibur; Beckton and Dickenson, USA) and data acquired using the Cell Quest software (Beckton and Dickenson, USA). Cell cycle analysis was performed using the Modfit program.

#### 2.6.3 Micronuclei formation

Air-dried slides containing acetic acid-methanol (1:3 V/V) fixed cells were stained with 2-aminophenylindoledihydrochloride (DAPI) (10 μg/mL in citric acid (0.01 M), disodium phosphate (0.45 M) buffer containing 0.05% Tween-20 detergent) as described earlier [18]. Slides were examined under fluorescence microscope. Cells containing micronuclei were counted from >1,000 cells by employing the criteria of Countrymen and Heddle [19]. The fraction of cells containing micronuclei, called the M-fraction (%) was calculated as follows:

where N_m _is the number of cells with micronuclei and N_t _is the total number of cells analyzed. Since, micronuclei formation is linked to cell proliferation, the micronuclei frequencies were normalized with respect to the cell numbers [14].

#### 2.6.4 Apoptosis

Detection and analysis of photodynamically induced apoptosis was performed by studying the morphological features, DNA content and changes in cell size, cytoskeleton structure associated with cells undergoing apoptosis.

##### Morphological studies

Morphologically, marked condensation and margination of chromatin, fragmentation of nuclei and cell shrinkage characterize apoptotic cells and a good correlation between these morphological changes and DNA ladder (one of the hallmarks of cells undergoing apoptosis) has been demonstrated [20]. Slides containing acetic acid-methanol (1:3 V/V) fixed and 2-aminophenylindoledihydrochloride (DAPI) stained cells were examined under fluorescence microscope using UV excitation filter and fluorescing nuclei were observed using a blue emission filter [18].

##### DNA analysis by flow-cytometry

Flow-cytometric measurements of cellular DNA content were performed with ethanol fixed cells. The presence of hypodiploid (sub G_0_/G_1_) population was taken to be indicative of the apoptotic cell population.

##### Measurements of light- scatter

Cells undergoing apoptosis generally shrink and are associated with changes in cytoskeletal structure, which is reflected in the alterations of light scatter. Therefore, treatment induced changes in forward and side scatter of incident light were investigated by collecting these signals in the list mode using cell-quest software (Beckton and Dickenson, USA). Analysis of light scatter was performed by off-line gating using appropriate windows created with untreated cells.

### 2.7 Statistical methods

Relationship between surviving fraction and energy (KJ) was quantified by modeling the data with a univariate linear regression analysis with energy being an independent variable and surviving fraction as dependent variable. Overall differences of mean relative proliferation among different treatment groups (1 μM, 5 μM, and control) as well as at each pre-specified hours (19, 30, 42 hours) were tested by using one-way analysis of variance (one-way ANOVA) with Bonferroni correction for pairwise group comparisons. For all the analysis, type-I error rate was set to 5% but multiple comparison was handled by using Bonferroni correction in which type-I error rate for pairwise group comparisons was set to 1.66%. A p-value of < 0.05 was considered statistically significant, if not stated otherwise due to Bonferroni correction for multiple comparisons. SAS v9.2 for windows (SAS Institute Inc., Cary, NC, USA) was used for statistical analysis of the data.

## 3. Results

### 3.1 Cellular uptake and sub-cellular localization of AlPcS_2_

**Figure 1:**
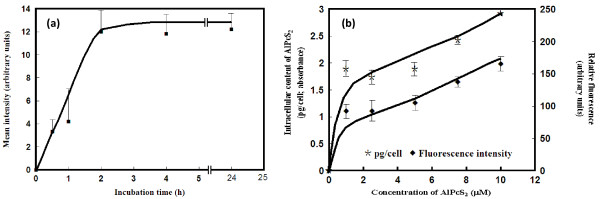
**(a). Cellular uptake of phthalocyanine as a function of time and concentration**. Uptake of AlPcS_2 _in exponentially growing glioma (BMG-1) cells as a function of incubation time at 37°C in HBSS containing the photosensitizer (1 μM) as determined by fluorescence image analysis (40-50 cells were examined from 2 experiments). The intensity of the background was subtracted from the values obtained for each cell from the same image. **(b) **Cellular uptake of AlPcS_2 _after incubation (2 h) of BMG-1 cells at different concentrations of AlPcS_2 _in HBSS. Measurements of absorbance and fluorescence were made in cell suspensions (n = 2).

Uptake kinetics of AlPcS_2 _following incubation of cells with AlPcS_2 _in HBSS for different time intervals showed rapid and linear increase in the accumulation of AlPcS_2 _in the first 2 h, prolonged incubation (up to 24 h), however, did not result in any further increase in the uptake (Figure 1a). HBSS was used to incubate the cells with AlPcS_2 _to reduce serum binding of AlPcS _2_. AlPcS_2 _is known to partition rapidly into the lipid bilayers and is transported inside the cells by the processes of diffusion and metabolically by endocytosis through binding with membrane proteins [21].

Estimation of cellular content of AlPcS_2 _after incubation for 2 h in different concentrations of AlPcS_2 _showed substantial uptake at 1 μM resulting in an average value of 1.9 ± 0.05 pg/cell, followed by a slower uptake up to 5 μM (Figure 1b). This pattern of uptake could result from aggregation of AlPcS_2 _and altered transport mechanism(s) at higher concentrations as reported earlier in studies of cellular uptake in a human nasopharyngeal cancer cell line [22].

### 3.2 Subcellular localization

#### 3.2.1 Effects of incubation time and concentration

**Figure 2:**
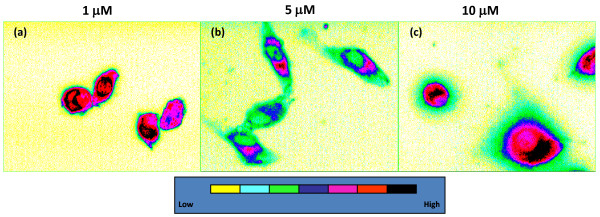
**[a-c]. Subcellular localization of phthalocyanine**. Concentration dependent localization of AlPcS_2 _(1-10 μM) in exponentially growing BMG-1 cells. Cells were incubated with the sensitizer for 2 h in HBSS and observed under fluorescence microscope. 40-50 cells for each treatment group were analyzed from 2-3 different experiments. Representative images at each concentration of AlPcS_2 _are shown after false coloring.

Immediately following incubation, AlPcS_2 _localized in the perinuclear region and no significant changes in the localization patterns were observed up to 4 h of incubation time (data not shown). Changes in localization as a function of concentration (1-10 μM) showed that AlPcS_2 _was distributed throughout the cytoplasm with intense fluorescence in the perinuclear regions up to a concentration of 2 μM, while a weak diffuse fluorescence was observed at higher concentrations (Figures 2a-c). Earlier studies with laser line-scanning confocal fluorescence microscopy have also shown that the intracellular fluorescence intensity of different phthalocyanine derivatives is dependent on the degree of aggregation as only monomer species exhibit fluorescence [21].

### 3.3 Photodynamic effects

Responses to different doses of AlPcS_2_-PDT were studied by investigating cell proliferation kinetics, cell-cycle perturbations, cytogenetic damage, apoptosis and clonogenic cell survival. The photodynamic dose was varied by changing light exposure and AlPcS_2 _concentrations during pre-incubation in HBSS. This incubation in HBSS for short intervals of time (2 h) did not compromise the survival.

#### 3.3.1 Clonogenic cell survival

Survival of glioma cells after damage induced by photo-irradiation in the presence of phthalocyanine was studied by the macrocolony assay, both as a function of light dose and concentration of AlPcS_2 _during pre-incubation.

**Figure 3:**
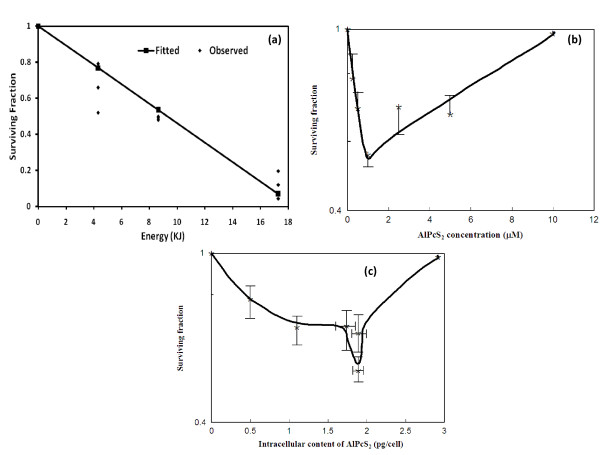
**[a-c]. Cell survival studied by colony forming assay after AlPcS_2_-PDT in BMG-1 cells**. Survival was investigated as a function of **(a) **light dose (AlPcS_2 _= 1 μM, 2 h), **(b) **AlPcS_2 _concentrations in the incubating medium and **(c) **different intracellular contents of AlPcS_2_. The intracellular content of AlPcS_2 _at 0.25 and 0.5 μM was calculated from Figure 1b. Irradiation was performed with red light at a total dose of 450 J/cm^2 ^after 2 h of post-irradiation incubation in HBSS (n = 3).

Relationship between surviving fraction and energy was quantified by modeling the data with a univariate linear regression analysis with energy being an independent variable and surviving fraction as dependent variable. As a result of fitting a univariate linear regression model, increasing energy significantly decreases the mean surviving fraction by 0.0538 (n = 12, MSE = 0.0090; Adjusted R-Square = 0.9692; p-value = 0.0001). The relationship between surviving fraction and energy can be quantified by the following regression equation "Surviving Fraction = 1-0.0538*Energy". Based on the analysis, a linear decrease was observed in the clonogenic cell survival of cells pre-incubated at 1 μM AlPcS_2 _for 2 h in HBSS after PDT with increasing light doses up to 1800 J/cm^2 ^(Figure 3a).

Experiments to study AlPcS_2 _concentration dependent dose-response (performed at the light dose of 450 J/cm^2^) showed a linear decrease in survival up to a concentration of 1 μM (Figure 3b). Interestingly, however, with further increase in AlPcS_2 _concentrations (2.5-10 μM), the surviving fraction did not decrease; instead a gradual increase was observed. At 10 μM, the survival was almost equal to the untreated cells (Figure 3b).

PDT induced cytotoxicity has been often correlated with the cellular uptake of the photosensitizer [23], the survival data plotted as a function of the cellular AlPcS_2 _content (Figure 3c) however, also showed a non-monotonic U-type dose-response.

To gain further insight, post-treatment proliferation kinetics of BMG-1 cells was studied.

#### 3.3.2 Growth Dynamics of Cell Populations

**Figure 4:**
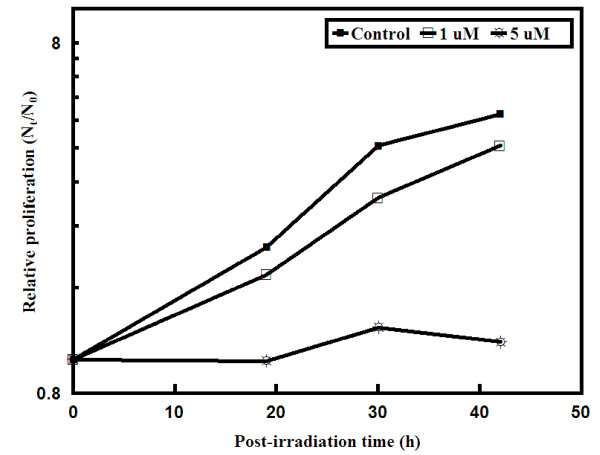
**Proliferation kinetics of BMG-1 cells following photodynamic treatment**. Cells were incubated with AlPcS_2 _for 2 h in HBSS and irradiated with red light (Power = 3 W/cm^2^; Light dose = 450 J/cm^2^). 2 h after irradiation, cells were allowed to grow in growth medium and both attached and detached cells were counted after different periods of growth (n = 3). Error bars are smaller than the size of the symbols and therefore are not visible.

Following photo-irradiation, significant retardations in the rates of cell proliferation were observed with increasing concentrations of AlPcS_2 _(Figure 4). At 1 μM AlPcS_2_, the population doubling time increased by nearly 4 h (from 19 to 23 h), while at 5 μM even one population doubling could not be observed after 42 h post treatment (Figure 4). Overall, regardless of the time, there were significant differences among treatment groups. Post-hoc pairwise comparisons indicated that mean relative proliferation was not significantly different between 1 μM *vs*. control but significant between 5 μM *vs*. control as well as 5 μM *vs*. 1 μM. The same analytical approach was carried out to test the differences in mean relative proliferation among different treatment groups (1 μM, 5 μM, and control) at each pre-specified times *i.e*. 19, 30, 42 hours. By taking into account the Bonferroni correction for multiple comparison with pairwise type-I error rate as 1.66%, there were no differences between 1 μM *vs*. control as well as between 5 μM *vs*. 1 μM at 19 hours and between 1 μM *vs*. control at 42 hours (p-values >1.66%). All other pairwise treatment differences were significant at 1.66% (Table 1). Table 1 provides the estimates of mean differences for each of the pairwise comparisons. Since all the mean difference estimates are negative, this also indicates that the first group listed in the pairwise comparison (1 or 5 μM) had lower estimated mean relative proliferation than the second treatment group (control or 1 μM).

**Table 1:** Testing of overall differences of mean relative proliferation among different treatment groups as well as at each pre-specified time by using one-way Analysis of Variance (one-way ANOVA) with Bonferroni correction for pairwise group comparisons.

	Pairwise Comparison	Mean Difference (Estimate [Standard Error])	P-value**
Overall			0.0001*

	1 μM vs. control	-0.8192 (0.4673)	0.0923

	5 μM vs. control	-2.6097 (0.4673)	0.0001

	5 μM vs. 1 μM	-1.7904 (0.4673)	0.0008

19 h			0.0001*

	1 μM vs. control	-0.3503 (0.3322)	0.3323

	5 μM vs. control	-1.1040 (0.3322)	0.0159

	5 μM vs. 1 μM	-0.7537 (0.3322)	0.0638

30 h			0.0001*

	1 μM vs. control	-1.183 (0.1116)	0.0001

	5 μM vs. control	-2.8440 (0.1116)	0.0001

	5 μM vs. 1 μM	-1.6610 (0.1116)	0.0001

42 h			0.0001*

	1 μM vs. control	-0.9243 (0.3034)	0.0226

	5 μM vs. control	-3.8810 (0.3034)	0.0001

	5 μM vs. 1 μM	-2.9567 (0.3034)	0.0001

* overall group differences calculated by F-test from one-way ANOVA

** all the p-values noted as "*" should be compared with 5% and all others with 1.66% due to pairwise comparisons with Bonferroni correction.

**Figure 5:**
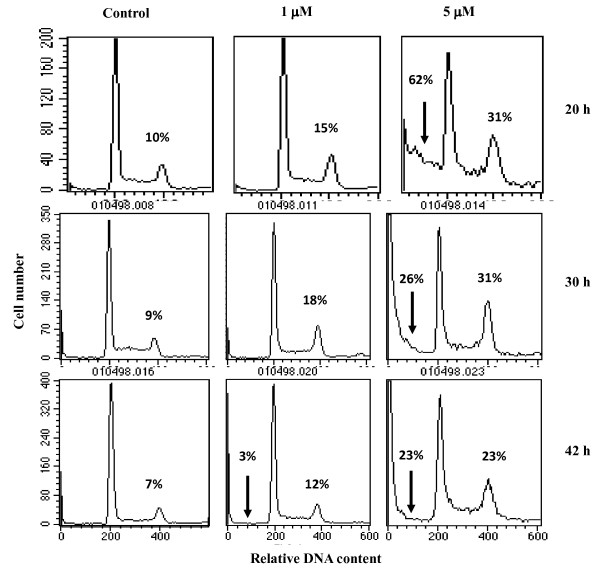
**Effects of photodynamic treatment on cell cycle distribution, induction of apoptosis and death**. Representative DNA histograms of PI stained BMG-1 cells measured by flow cytometry are shown. Cells were incubated for 2 h with AlPcS_2 _in HBSS, irradiated with red light at a light dose of 450 J/cm^2 ^and incubated for different time periods in growth medium before staining.

Cell-cycle analysis carried out from the flow-cytometric measurements of DNA content revealed that cells accumulated in G_2_+M phase indicating that the cell progression through the G_2 _phase was blocked after AlPcS_2_-PDT, particularly at the higher AlPcS_2 _concentration (Figure 5). Similar G_2 _block has also been reported in human chronic myelogenous leukemia cells after AlPcS photosensitization under a wide range of light dose and pre-incubation times [24].

Frequency of non adherent and floating cells in the culture increased after AlPcS_2_-PDT only in cells incubated at the higher AlPcS_2 _concentration (5 μM), while no significant change was observed at 1 μM AlPcS_2 _(Table 2). These observations are in line with PDT induced alterations in the cell adhesion characteristics, linked to membrane damage [25,26] and cytoskeleton [27,28].

**Table 2:** Comparison of photodynamic effects in BMG-1 cells pre-incubated with different concentrations of AlPcS_2_.

Endpoint^a^	Time after Photo- irradiation	0.0 μM	1.0 μM	5.0 μM	10.0 μM
Intracellular content of AlPcS_2 _(pg/cell) (absorbance)	2 h	0.0	1.9 ± 0.05	1.9 ± 0.06	2.9 ± 0.05

Relative fluorescence intensity	2 h	0.0	92 ± 13.0	105 ± 10.0	165 ± 9.0

Fluorescence Distribution	2 h	-	Perinuclear and Granular in cytoplasm	Diffuse in cytoplasm	Diffuse in cytoplasm but membrane damage in some cells

Clonogenic Survival	240 h	1.0 ± 0.0	0.53 ± 0.07	0.65 ± 0.1	0.98 ± 0.22

Proliferation Index^b^	42 h	5.6 ± 0.4	3.5 ± 0.5	1.0 ± 0.4	ND^c^

G_2_+M (%)	42 h	7.0 ± 2.0	12.0 ± 2.0	23.0 ± 0.8	ND

Detached Cells (%)	42 h	19.0 ± 2.8	15.0 ± 0.3	30.0 ± 8.0	ND

Small cells (%)	42 h	4.0 ± 0.1	24.0 ± 0.2	57.0 ± 0.1	ND

Sub G_0_/G_1 _(%)	42 h	0.0	3.0 ± 0.2	23.0 ± 10.0	ND

M. F. (%)	42 h	1.6 ± 0.2	1.5 ± 0.1	1.7 ± 0.5	ND

^a^Endpoints are summarized as mean ± standard deviation by groups.

^b^N_t_/N_0 _Where Nt is the cell number at 42 h and N0 is the cell numbers at the time of irradiation.

^c^ND- not determined.

It is pertinent to note that since almost equal amounts of cellular AlPcS_2 _accumulated at both the concentrations (1 and 5 μM), the PDT-induced differences in the proliferation kinetics observed here, must arise from the concentration dependent differences in the patterns of sub-cellular distribution of the photosensitizer.

#### 3.3.3 Apoptotic Cell Death

An analysis of DNA flow-cytograms (Figure 5) showed significant increases in the sub G_0 _fraction of cells indicating considerable DNA fragmentation (apoptotic and necrotic death) after PDT at higher concentration of AlPcS_2_, while at 1 μM AlPcS_2_, little differences as compared to untreated controls were observed. In contrast, a reduction in forward angle light scatter implying a reduction in the cell size (measured from 20-42 h after PDT) could be observed to a significant extent even at 1 μM AlPcS_2 _(data at 42 h shown in Table 2). Rounded cells with nuclei showing an apoptosis like morphology were also observed microscopically at both the concentrations of AlPcS_2_, similar to the observations reported in HeLa cells with ZnPC [29].

Since DNA fragmentation is a late stage event in the apoptotic process, the observed differences may indicate concentration dependent variations in the apoptotic pathways.

#### 3.3.4 Cytogenetic damage

*In vitro *studies on DNA solutions have indicated that metallo-phthalocyanines can induce significant numbers of DNA strand-breaks [30]. Single strand DNA breaks and mutagenicity induced by photodynamic action of aluminum phthalocyanine have been detected in yeast [31]. In mammalian cells frequency of mutations induced by AlPc-PDT has been shown to be dependent on the p53 status and cellular repair capacities[32]. Present studies, however, demonstrate lack of significant AlPcS_2_-PDT induced cytogenetic damage as studied by monitoring the induction of micronuclei which arise mainly from DNA double strand breaks and chromosomal aberrations in the post-mitotic cells (Table 2). These observations are in agreement with earlier studies on the inability of phthalocyanine photosensitization to induce mutagenesis and micronuclei formation[33,34].

## 4. Discussion

Present studies demonstrated important differences between the AlPcS_2_-PDT induced changes in the proliferation kinetics and clonogenic survival of glioma cells pre-incubated under different concentrations of AlPcS_2 _(summarized in Table 2). The cellular uptake as a function of extracellular AlPcS_2 _concentrations was observed to be biphasic; an initial rapid rate at lower concentrations was followed by a slower uptake with increasing concentration of the sensitizer. The subcellular distribution of AlPcS_2 _also varied with its extracellular concentration. While cell proliferation kinetics showed a monotonic increase in the photodynamic effects with increasing AlPcS_2 _concentrations (Figures 4 &5), colony -forming assay showed a U-type dose-response with an initial increase in cell death followed by enhanced survival at higher levels of AlPcS_2 _(Figure 3). This appears to be exceptional since, at constant oxygen environment, the photodynamic effects are generally observed to increase monotonously on increasing either the light dose or/and cellular content of the sensitizer [13,23]. However, several examples of non-monotonic dose-response relationships for a variety of end-points have been demonstrated in the field of toxicology and explained on the basis of complex interactions of biological processes involved [35,36]. Physico-chemical and biological processes that may underlie the concentration dependent photodynamic effects observed in the present studies with AlPcS as the photosensitizer have implications for designing therapeutic protocols.

### 4.1 Physico-chemical interactions of the photosensitizer, its cellular uptake and sub-cellular localization

AlPcS behaves like a typical amphiphile with charged substituents located at the membrane/buffer interface and the non-polar portion of the molecule in contact with the hydrophobic lipid chains [37]. Such a dye-membrane interaction would allow the charged sulphonated phthalocyanine to bind to membrane transport proteins and to enter the cell cytoplasm preferably by the processes of endocytosis, while the diffusion processes provide only a small contribution [21]. At higher concentrations, all the sites on the surface receptor proteins could be occupied resulting in a saturation of cellular uptake of AlPcS. Indeed, pre-incubation of the cells at AlPcS_2 _concentrations between 1-5 μM resulted in the same cellular content of the photosensitizer (Figure 1b). Many of the water soluble PcS compounds are also susceptible to formation of dimers or aggregates [38,39]. At high concentrations of AlPcS_2_, higher aggregates may be formed and additional transport mechanisms could be induced. The relative fluorescence intensity, monitored by whole cell spectroscopy, in BMG- 1 cells incubated at 10 μM AlPcS_2 _was about 50 times and 100 times less than the RFI in HBSS and methanol respectively (data not shown) indicating the aggregation of AlPcS_2 _at higher concentrations. Present observations are in agreement with studies in V-79 cells where it has been shown that intracellular fluorescence intensity of various phthalocyanine derivatives vary with their aggregation capacity [21].

The sub-cellular localization is one of the key factors that determine the type of photodynamic effects [40]. Interestingly, in the present studies, the intracellular localization of AlPcS_2 _was observed to be dependent on its extracellular concentrations. It was localized in a granular fashion throughout the cytoplasm with intense fluorescence in the perinuclear region at lower concentrations while at higher concentrations AlPcS_2 _fluorescence was weak and diffused (Figure 2). Possibly, at lower concentrations, AlPcS_2 _was localized to more sensitive targets leading to greater photodynamic cell killing than at higher concentrations.

### 4.2 Photophysical and photochemical reactions underlying production of ROS

A number of competing photophysical and photochemical reactions depending on the intracellular microenvironment of AlPcS and its molecular density may influence its photodynamic efficacy and therefore the outcome of therapy.

The decrease in photodynamic cytotoxicity induced by AlPcS_2 _at higher concentrations (>1 μM) could also be due to the intracellular presence of photodynamically inactive species like aggregates [41]. Although, significant changes in the fluorescence spectra (peak asymmetry or broadening) indicative of aggregation were not observed at different concentrations of AlPcS_2_, the RFI monitored by whole cell spectroscopy at 10 μM AlPcS_2 _was many folds less than the RFI in HBSS and methanol indicating the aggregation of AlPcS_2 _at higher concentrations. These observations suggest that AlPcS_2 _was not aggregated in HBSS before uptake but was aggregated once it was taken up by the cells. The present results are similar to the observations made earlier in V79 cells, where cells incubated with 1 μM and 3 μM of AlPcS_1 _were more sensitive per quantum of fluorescence than the cells incubated with 10 μM indicating that all the sulphonated AlPc derivatives inside the cells are partly aggregated, the degree of aggregation being dependent on lipophilicity [42]. The phototoxicity of ClAlPcS_4_^4- ^(commercially available) and pAlPcS_4_^4- ^(isolated by HPLC fractionation) has also been reported to reduce with increasing concentrations of the sensitizer due to aggregation at the higher concentrations in a human nasopharyngeal cancer cell line (KB) [22]. It has been hypothesized that only the monomeric forms of AlPcS_2 _fluoresce and have a detectable triplet state and also involved in the production of singlet oxygen [43].

High intracellular concentrations of the sensitizer may also result in an inner filtering of light contributing to the reduced photodynamic efficiency. Phthalocyanines have been shown to be highly efficient quenchers of singlet oxygen [44]. It is probable that a high density of the AlPcS molecules enhances photo-bleaching and singlet oxygen quenching. Further, the dependency of fluorescence bleaching on the environment of dye has also been reported [45,46]. Therefore, different localizations of AlPcS_2 _at different concentrations could result in varying amounts of photobleaching leading to reduced production of ROS at high concentrations.

### 4.3 Cellular responses to PDT induced damage

It is intriguing that the effects of AlPcS_2_-PDT on the macrocolony assay (Figures 3b and 3c) appear different from the proliferation kinetics. While, the proliferation kinetics parameters investigated were measured in monolayer cell cultures at high cell densities, colony-forming assays were performed after plating at low cell density. Cell density dependent cell to cell interaction mediated death and recovery processes (bystander effects) are, however, unlikely to contribute significantly since the cell density was nearly identical for all the groups in colony -forming assay. Post-treatment time at which observations are made could be important. In contrast to the proliferation kinetics which were studied during the first 42 hours post-treatment (about 2 cell cycles), cell survival using colony-forming assay was measured after 10-12 days (time required for formation of visible macro-colonies containing at least 50 cells *viz*. after completion of 8-10 cell-cycles depending on the division delay), and would, thus, include modifications induced by the late repair and death processes. Also stress-induced premature senescence (SIPS), after sub-lethal oxidative damage [47,48], could reduce the number of observed macro-colonies.

Damage related division delay that purportedly facilitates cellular recovery processes on account of checkpoints either before the DNA synthesis (G_1_-S transition) or mitotic division (G_2 _block) has been shown to enhance cell survival following damage caused by many physical and chemical agents [49]. A significant G_2_+M block, observed at 5 μM (Figure 5) supports this proposition. A decrease in the fraction of small cells (indicative of severe structural damage) observed at 62 h after treatment (Table 2 and data not shown) lends further support to the contribution of cellular recovery processes, that may be triggered beyond a certain threshold level of damage that facilitate cells to recover from potentially lethal damage.

The results obtained in the present studies indicate that at least two pathways may contribute competitively or additively to phototoxicity. One that manifests early (in less than one or two cell-cycle after treatment) (Figure 5), while the other is delayed where cells die with successive divisions similar to mitotic death induced by ionizing radiation. Although, the early cell death increased with increasing concentrations of the sensitizer, it appears that its contribution to the overall clonogenic survival is not very significant (Figure 5). The predominant death appears to be the delayed type, possibly the induced lesions responsible for this mode of cell death may be reduced at high concentrations of the sensitizer. The fractions of floaters (cells detached from the dishes representing degenerating cells) observed under these conditions also lend support to this possibility (Figure 5 and Table 2). While, the floaters in control and 1 μM group may be due to increase in cell proliferation (Figure 4 and Table 2), in the absence of significant increase in population growth at 5 μM, the floaters were clearly due to the damage rather than due to increased cell density.

Although, AlPcS_2_-PDT resulted in classical features of apoptosis *viz*. induction of sub G_0_/G_1 _population only at 5 μM AlPcS_2 _(Figure 5), a reduction in forward scatter indicative of cell shrinkage (one of the features of apoptotic cells) could be observed even at a concentration of 1 μM (Table 2). Interestingly, rounded cells with nuclei showing an apoptosis like morphology were observed with both the AlPcS_2 _concentrations, similar to the observations reported in HeLa cells with ZnPC [29]. This morphology has been attributed to photodamage to microtubular (MT) network since it has been shown that MT disruption is involved in apoptosis [50,51]. Depolymerization of tubulin may be caused by an increase in PDT induced intracellular calcium (Ca^2+^) [52]. Role of calcium in photofrin and phthalocyanine mediated photohemolysis and apoptosis in rabbit red blood cells, human squamous carcinoma cell line and rat bladder RR1022 epithelial cells has been reported [12,53,54]. Since, the photosensitization reactions depend on the sub-cellular location of the sensitizer and AlPcS_2 _distributed diffusely in the cytoplasm with intense perinuclear fluorescence, damage to cytoskeletal elements could be one of the factors triggering apoptosis. This could also contribute to reduction in initial rate of proliferation of cells pre-incubated at higher concentrations of AlPcS_2_. However, induction of G_2_-block to a greater extent may allow the remaining cells to recover from the potentially lethal lesions under these conditions and contribute to a higher clonogenic survival.

## 5. Conclusions

Results of the present investigations imply that the AlPcS_2_-PDT efficacy under certain circumstances may not increase monotonically with the increase in photodynamic dose varied by changing the concentration of the photosensitizer. Based on the present results, we hypothesize that the non-monotonic photodynamic effects could arise due to multiple reasons including (a) concentration dependent changes in physico-chemical properties of AlPcS_2 _due to varying degrees of aggregation leading to different patterns of cellular transport and intracellular localization, (b) complex interactions between photobleaching and singlet oxygen quenching at high intracellular densities of AlPcS_2 _and its aggregates and (c) competitions between cellular proliferation, cellular repair/misrepair and cell death pathways following induction of photodynamic lesions. Detailed further studies are warranted to verify this hypothesis and to elucidate precise mechanisms underlying the phenomena observed in the present studies. Most importantly, these results strongly suggest that the therapeutic efficacy of PDT need not always be higher with higher PDT doses achieved either by large sensitizer and/or light doses. Further the *in vivo *responses are likely to be confounded by other factors related to tumor physiology as well as systemic effects. Therefore, predictive assays using appropriate *in vitro *models that better represent environmental factors prevailing in tumors will be helpful in designing most effective therapy for a given tumor.

## 6. List of abbreviations

AlPcS_2_: disulphonated aluminum phthalocyanine; BMG-1: human glioma cell line; DAPI: 2-aminophenylindoledihydrochloride; HpD: hematoporphyrin derivative; PDT: photodynamic treatment; PI: propidium iodide; RFI: relative fluorescence intensity; ROI: region of interest; ROS: reactive oxygen species.

## 7. Competing interests

The authors declare that they have no competing interests.

## 8. Authors' contributions

SG carried out all the experiments, acquired, analyzed and interpreted the data and drafted the manuscript. BSD participated in the design of the study, made contributions to acquisition, analysis and interpretation of data and helped to draft the manuscript. KM helped in the interpretation of the data on uptake and localization of phthalocyanine and drafting of the manuscript. VJ conceived the study and participated in its design and coordination and helped in interpretation of data and drafting the manuscript. TKS made contribution to statistical analysis and interpretation of data and helped to draft the revised manuscript.

The final manuscript is read and approved by all the authors.
